# Supplementary material for: First description of Nodding Syndrome in the Central African Republic
Source: PLoS Negl Trop Dis. 2021 Jun 18;15(6):e0009430. doi: 10.1371/journal.pntd.0009430 (PMC8244846; doi:10.1371/journal.pntd.0009430)
Supplement: S1 File — (PDF) [file pntd.0009430.s001.pdf]

FICHE DE DEPISTAGE DU NODDING SYNDROME ET DE L'EPILEPSIE

NOM de l'enquêteur: ____ DATE : |____/____/____|

Identifiant du ménage : ________

Quartier : ____

Une photo de la maison a été prise ? ☐ Oui ☐ Non

Sinon, pourquoi ? ____

Qui répond aux questions? ☐ Chef de famille ☐ Autre , préciser _______________________

Nom de celui qui répond : ____

Tel : ____

Combien de personnes vivent dans la maison ? __________

Combien ont entre 6 et 9 ans ? ________

N°	Question	Réponse	Aller à	Code
**Questionnaire : Dépistage du Nodding Syndrome et épilepsie**				
**Le sujet enquêté a-t-il déjà présenté ?**				
Q1	Une crise de type hochement de tête (chutes répétitives et involontaires de la tête sur la poitrine) par perte de tonicité des muscles du cou? Ou tout autre mouvement anormal, incontrôlé et inquiétant de la tête faisant penser à un relâchement des muscles du cou ?	01. Oui<br>02. Non<br>98. Ne sais pas<br>99. Ne souhaite pas répondre		|__|__|
Q2	Des chutes d'installation brutale et de durée brève dues à une faiblesse musculaire généralisée?	01. Oui<br>02. Non<br>98. Ne sais pas		|__|__|
Q3	Perte (s) de connaissance et/ou perte d'urine et/ou émission de bave ?	01. Oui<br>02. Non		|__|__|
Q4	Absence(s) ou perte(s) de contact avec l'entourage de début brutal et de durée brève?	01. Oui<br>02. Non<br>98. Ne sais pas		|__|__|
Q5	Secousses ou mouvements anormaux incontrôlables au niveau d'un ou des membres (convulsions), de début soudain et d'une durée de quelques minutes ?	01. Oui<br>02. Non<br>98. Ne sais pas		|__|__|
Q6	Apparition brutale et de durée brève de sensations corporelles étranges d'hallucinations ou d'illusions visuelles, auditives ou olfactives ?	01. Oui<br>02. Non<br>98. Ne sais pas		|__|__|
Q7	A-t-on déjà dit au sujet qu'il était épileptique ou qu'il avait déjà présenté des crises d'épilepsie ou de hochement de la tête ?	01. Oui<br>02. Non<br>98. Ne sais pas		|__|__|
Q8	Des symptômes psychiatriques (à type d'étourdissement, divagation, déambulement, violence etc.) à la suite d'une de ces crises ?	01. Oui<br>02. Non<br>98. Ne sais pas		|__|__|

Liste des cas suspects de Nodding syndrome ou d'épilepsie

N°	Nom et Prénoms	Date de naissance	Lieu de naissance	NS	EP	OV
1		|__|__| / |__|__| / |__|__| Jj mm aa				
2		|__|__| / |__|__| / |__|__| Jj mm aa				
3		|__|__| / |__|__| / |__|__| Jj mm aa				
4		|__|__| / |__|__| / |__|__| Jj mm aa				
5		|__|__| / |__|__| / |__|__| Jj mm aa				
6		|__|__| / |__|__| / |__|__| Jj mm aa				
