# Supplementary material for: First description of Nodding Syndrome in the Central African Republic
Source: PLoS Negl Trop Dis. 2021 Jun 18;15(6):e0009430. doi: 10.1371/journal.pntd.0009430 (PMC8244846; doi:10.1371/journal.pntd.0009430)
Supplement: S2 File — (PDF) [file pntd.0009430.s002.pdf]

Questionnaire confirmation épilepsie

NOM de l'enquêteur: ____ DATE : |______/______/______|

☐ Neurologue Identifiant : |______/______/______|

1. Renseignements généraux

1.1. Le répondant est-il le participant lui-même ? ☐ OUI ☐ NON

Si NON, quelle est la relation entre le participant et le répondant ? ______________________________

1.2. Un traducteur a-t-il été nécessaire ? ☐ OUI ☐ NON

1.3. Depuis quand le participant vit-il dans le village? ☐< 1 an ☐ > 1an ☐ Ne sait pas

Si plus de 1 an, nombre d'années : ___________années

Si la personne vivait avant dans un autre village, lequel? ______________________________

2. Caractéristiques socio-démographiques

2.1. Age : _______________

2.2. Sexe : ☐ M ☐ F

2.3. Poids (kg): _________, _____kg

2.4. Taille (cm) : ____________ cm

2.5. Niveau scolaire : ☐Aucun ☐Primaire ☐Secondaire ☐Supérieur

2.6. Activité principale du participant: ☐ aucune si enfant ☐ agriculture ☐ pêche ☐ chasse

2.7. ☐ élevage ☐ commerce ☐ artisan ☐ autre, spécifier : _________________

3. Notion de la prise de Mectizan

3.1. Est-ce que le participant a déjà pris Mectizan (par voie orale) ☐Oui ☐ Non ☐ NSP

Si oui, combien de fois? __________ nombre de fois

3.2. Est-ce que le participant a déjà pris Mectizan (par voie orale) avant le début de l'épilepsie ?

☐ OUI ☐ NON ☐ Ne sait pas

3.3. Au cours de ces deux dernières années, le participant a-t-il pris du Mectizan ? ☐ OUI ☐ NON

3.4. Quand le participant a-t-il pris la dernière fois Mectizan? Année 20____ Mois_________

4. Antécédents concernant la naissance/enfance du participant enquêté

4.1. La mère présente pour répondre aux questions ? ☐ Oui ☐ Non

4.2. Lieu de naissance du participant enquêté ?

☐ Domicile ☐ Centre de Santé ☐ Hôpital ☐ Autre ☐ NSP

4.3. Accouchement : ☐ normal ☐ accouchement difficile/ventouse/forceps ☐ par césarienne

4.4. Le participant enquêté est-il né prématuré ? ☐ Oui ☐ Non ☐ NSP

4.5. Est-ce que l'enfant a crié à la naissance ☐ Oui ☐ Non ☐ NSP

4.6. Age de début des symptômes de l'épilepsie : _______________

4.7. Est-ce qu'avant l'épilepsie, le participant a souffert de :

☐traumatisme crânien? ☐ encéphalite/méningite? ☐ neuro-paludisme?

☐ rougeole ☐ maladie du sommeil ? ☐ autre maladie grave ☐ NSP

4.8. si autre maladie, spécifier ____

4.9. Convulsions fébriles dans l'enfance (0-10ans). ☐ OUI ☐ NON ☐ NSP

Si oui, à quel âge les convulsions fébriles ont débuté ? _________________________

5. Caractéristiques des crises épileptiques

5.1. Perte de connaissance et/ou convulsions?

☐ Jamais ☐ Une fois ☐ Deux fois ou plus ☐NSP

5.2. Perte(s) de connaissance avec perte(s) d'urine ?
☐ OUI ☐ NON ☐ NSP

5.3. Perte(s) de connaissance avec émission de bave et/ou morsure de langue?
☐ OUI ☐ NON ☐ NSP

5.4. Absence(s) ou perte(s) de contact brève avec l'entourage (participant ne répond pas, de début brutal et de durée brève)?
☐ OUI ☐ NON ☐ Ne sait pas

5.5. Épisodes de hochement de tête (HT) ?
☐ OUI ☐ NON ☐ NE SAIT PAS

5.6. A quel âge les hochements de tête ont-ils commencé ? _________________

5.7. Est-ce que la personne présente toujours de hochements de tête ?
☐ Oui ☐ Non ☐ NSP
Si non, depuis quel âge les hochements de tête ont-ils disparu? _________________________________
Préciser la cause de la disparition du HT : ☐ spontanément ☐ traitement antiépileptique ☐ NSP
Spécifier le traitement : ____

5.8. Secousses ou mouvements anormaux incontrôlables d'un ou des membres (convulsions, de début soudain et
5.9. d'une durée de quelques minutes) ?
☐ Oui ☐ Non ☐ NSP

5.10. Apparition brutale et de durée brève de sensations corporelles étranges, d'hallucinations ou d'illusions
5.11. visuelles, auditives ou olfactives?
☐ Oui ☐ Non ☐ NSP

5.12. Estimation de la durée de la crise :__________________minutes

6. Antécédents de crises d'épilepsie

6.1. A-t-on déjà dit à la personne qu'elle était épileptique ou qu'il avait déjà fait des crises d'épilepsie ?
☐ Oui ☐ Non ☐ NSP

6.2. Le participant a-t-il présenté une crise dans les 5 dernières années ? ☐ Oui ☐ Non ☐ NSP

6.3. Le participant a-t-il présenté une crise la dernière année ☐ Oui ☐ Non ☐ NSP

6.4. Le participant a-t-il présenté une crise le dernier mois ☐ Oui ☐ Non ☐ NSP

6.5. Combien de crises d'épilepsie le participant a-t-il présenté par an? _________ fois/an.
Si le participant à présente :
plus de 12 crises d'épilepsie par an, alors, préciser le nombre de crise par mois : _________ fois/mois
plus de 30 crises d'épilepsie par mois, alors, préciser le nombre de crise par jour : _________ fois/jour

7. Type d'épilepsie

7.1. Crises généralisées : ☐ OUI ☐ NON
Si oui, ☐ tonico-cloniques ☐ atoniques ☐ autre, spécifier_______________________________

7.2. Absences ☐ OUI ☐ NON

7.3. Crises avec début focale sans de perte de connaissance: ☐ Oui ☐ Non
Si oui, ☐ motrices ☐ sensorielles

7.4. Crises avec début focal avec perte de connaissance : ☐ Oui ☐ Non
Si oui : ☐ motrices ☐ sensorielles

7.5. Crise avec début focal secondairement bilatéralement tonico-clonique : ☐ Oui ☐ Non

7.6. Hochement de tête (avec/sans altération de l'état de conscience) ☐ Oui ☐ Non ☐ NSP
Si oui, est-ce que durant le hochement la personne :
☐ ne répond pas aux questions ☐ arrête de manger ☐ tombe/convulse ☐ reste lucide

7.7. Quel facteur peut déclencher la crise (hochement de tête ou autre forme d'épilepsie)?
(plusieurs réponses possibles) : ☐pas de facteur spécifique ☐ surtout au réveille ☐la nuit ☐le froid
☐le repas ☐les orages ☐ Ne sait pas ☐autre, à spécifier:____

8. Développement psycho-moteur du participant (comparativement aux enfants du même âge)

8.1. AVANT le début de l'épilepsie ?

8.1.1. Capacité de marcher ? ☐ tôt ☐ même âge ☐ plus tard ☐ nsp

8.1.2. Capacité de parler avec des phrases ?☐ tôt ☐ même âge ☐ plus tard ☐ nsp

8.1.3. Est-ce que l'enfant a grandi ☐ plus vite ☐ normalement ☐ moins vite ☐ nsp

8.2. APRES début de l'épilepsie ?

8.2.1. Capacité de marcher ? ☐ tôt ☐ même âge ☐ plus tard ☐ nsp

8.2.2. Capacité de parler avec des phrases ?☐ tôt ☐ même âge ☐ plus tard ☐ nsp

8.2.3. Est-ce que l'enfant a grandi ☐ plus vite ☐ normalement ☐ moins vite ☐ nsp

8.3. Est-ce que durant l'année précédant la première crise d'épilepsie la personne présentait déjà une régression des acquisitions psycho-motrices? ☐Oui ☐ Non ☐ nsp

Si oui, spécifier :____

9. Traitements

9.1. Prenez-vous des médicaments MODERNES contre l'épilepsie?

☐ Jamais ☐ De façon irrégulière ☐ Après chaque crise ☐ Continuellement.

Si prise d'antiépileptiques modernes, préciser lesquels? ____________________________________

9.2. Prenez-vous des médicaments TRADITIONNELS contre l'épilepsie?

☐ Jamais ☐ De façon irrégulière ☐ Après chaque crise ☐ Continuellement.

10. Antécédent familiaux

10.1. Est-ce qu'il y a d'autres personnes avec épilepsie dans la famille ☐Oui ☐Non ☐ nsp

Si oui, préciser : ☐ père ☐ mère ☐ frère ou sœur : préciser le nombre : __________

10.2. Le participant enquêté a-t-il un jumeau ou une jumelle identique ? ☐ Oui ☐Non ☐ nsp

10.3. Si oui, est-ce que le jumeau souffre d'épilepsie ? ☐ OUI ☐ NON ☐ nsp

**11. Examen clinique**		
11.1. État général	☐Bon ☐ modérément altéré ☐ mauvais	
11.2. Vision normale	☐OUI	☐NON
11.3. Œil vitreux	☐OUI	☐NON
11.4. Aveugle?	☐Un œil ☐ 2 yeux ☐NON	
11.5. Nombre de cicatrices de brulures________________________________		
11.6. Rétractions dues aux brulures	☐OUI	☐NON
11.7. Lésions traumatiques dus aux crises	☐OUI	☐NON
11.8. Nombre de lésions traumatiques du a aux crises	________________	
11.9. Papules prurigineuses	☐OUI	☐NON
11.10. Peau de léopard	☐OUI	☐NON
11.11. Peau de lézard	☐OUI	☐NON
11.12. Démangeaisons / prurit	☐OUI	☐NON
11.13. Peau normale	☐OUI	☐NON
11.14. Nodules sous cutanés palpables	☐OUI	☐NON
11.15. Lymphoedeme	☐oui	☐non
11.16. Candidose buccale	☐oui	☐non
11.17. Signes de morsures de langue	☐oui	☐non
11.18. Ganglions cervicaux anormaux (>1cm)	☐oui	☐non
11.19. Adolescent/adulte qui ressemble a un enfant	☐oui	☐non
11.20. Déformation corporelle: ☐ thoracique ☐colonne vertébrale ☐ visage ☐ si déformations, spécifier :____		
**12. Examen neurologique**		

12.1. Conscience ☐ normale ☐ altérée

12.2. Trouble mental ? ☐ Oui ☐ Non

Si oui, préciser l'importance :

☐ Peu important (ne dérange pas les activités)

☐ Modérément important (dérange les activités mais encore actif)

☐ Important (empêche d'exercer les activités habituelles)

12.3. Est-ce que avant le développement de l'épilepsie la personne présentait déjà, ces troubles mentaux ☐ Oui ☐ Non

12.4. Si oui, préciser____

12.5. La marche est-elle normale ?

☐ Oui ☐ base élargie ☐ spastique ☐ hémiplégique

12.6. Faiblesse musculaire ? ☐ Non ☐ généralisée ☐ localisée

12.7. Paralysie? ☐ Non ☐ Oui, spécifier____

12.8. Contractures ? ☐ Non ☐ Oui

12.9. Conclusion examen neurologique : ☐ Normal ☐ Anormal, spécifier________________________

13. Diagnostic

☐ Epilepsie, sans hochement de tête ☐ Syndrome du hochement de tète

☐ Hochement de tête et autre forme d'épilepsie ☐ Non-épileptique

☐ Autre diagnostic à spécifier :____

PRELEVEMENTS / TESTS

14. Test biologique

Résultat du test OV16 : ☐ Négatif ☐ Positif ☐ pas fait

Signature MD/personnel santé **Date : |__|__| /|__|__| /|__|__|__|__|**
