# Supplementary material for: First description of Nodding Syndrome in the Central African Republic
Source: PLoS Negl Trop Dis. 2021 Jun 18;15(6):e0009430. doi: 10.1371/journal.pntd.0009430 (PMC8244846; doi:10.1371/journal.pntd.0009430)
Supplement: S3 File — (PDF) [file pntd.0009430.s003.pdf]

# Nodding Syndrome en RCA 2019 (Confirmation)

CONFIRMATION DU NODDING SYNDROME

**Nom de l'enquêteur**

- ◯ Pascal MBELESSO
- ◯ Salvatore METANMO

**Spécialité de l'enquêteur**

- ◯ Neurologue
- ◯ Medecin généraliste

**Date**

yyyy-mm-dd

**Identification du patient**

**1-1 Nom(s) et Prénom(s) du Patient**

**1-2 Date de naissance**

*Remarque : Si la date exacte est inconnue, marquer 2020-01-01 et mettre l'âge approximatif*

yyyy-mm-dd

**1-3 Âge**

**1-4 Lieu de naissance (ville ou village)**

*Marquer Inconnu si ne sait pas*

**1-5 Sexe**

- ◯ Masculin
- ◯ Féminin

**1-6 Qui répond aux questions**

- ◯ Enquêté (e)
- ◯ Mère
- ◯ Père
- ◯ Frère ou sœur
- ◯ Autre

**1-7 Quel est le lien de consanguinité avec l'autre le cas échéant ?**

______________________________

**1-8 A quel âge les chutes/hochements de tête sur la poitrine ont-ils commencé?**
*Mettre l'âge en mois, si inconnu mettre 999*

______________

**1-9 Est que qu'avant la chute de la tête, le sujet a souffert de :**

- ☐ Traumatisme crânien
- ☐ Encéphalite/méningite
- ☐ Neuro-paludisme
- ☐ Rougeole
- ☐ Onchocercose (cécité des rivières)
- ☐ Autre maladie grave
- ☐ Aucune
- ☐ Inconnu

**1-10 Si autre maladie grave, spécifier**

______________________________

**1-11 Si pathologie avant la chute de la tête, combien de mois avant ?**
*Si inconnu mettre 999*

______________

**1-12 Est-ce que le sujet présente toujours des chute/hochements de tête sur la poitrine ?**

- ◯ Oui
- ◯ Non
- ◯ Inconnu

**1-13 Si non, depuis quel âge les chutes/hochements de tête ont-ils disparu?**
*Mettre l'âge en mois, si inconnu mettre 999*

______________

**1-14 Préciser la cause de disparition des chutes/hochements de tête**

- Spontanément
- Traitement anti-épileptique
- Traitement traditionnel
- Autre
- Inconnu

**1-15 Si autre, préciser**

---

**1-16 Est-ce que le sujet présente aussi des crises épileptiques( convulsives) ?**

- Oui
- Non
- Inconnu

Si le sujet présente des crises épileptiques (convulsives), les décrire

**1-17 Ont-elles commencé avant ou après les chutes/hochements de tête ?**

- Avant
- Apres
- Inconnu

**1-18 Perte de connaissance et/ou convulsions?**

- Jamais
- Une fois
- Deux fois ou plus
- Inconnu

**1-19 Perte(s) de connaissance avec perte(s) d'urine ?**

- Oui
- Non
- Inconnu

**1-20 Perte(s) de connaissance avec émission de bave et/ou morsure de langue?**

- Oui
- Non
- Inconnu

**1-21 Absence(s) ou perte(s) de contact brève avec l'entourage (participant ne répond pas, de début brutal et de durée brève)?**

- Oui
- Non
- Inconnu

**1-22 Secousses ou mouvements anormaux incontrôlables d'un ou des membres (convulsions, de début soudain et d'une durée de quelques minutes) ?**

- Oui
- Non
- Inconnu

**1-23 Apparition brutale et de durée brève de sensations corporelles étranges, d'hallucinations ou d'illusions visuelles, auditives ou olfactives?**

- Oui
- Non
- Inconnu

**1-24 Estimation de la durée de la crise**

*En minutes, mettre si 99 si inconnue*

____________________

Préciser le type d'épilepsie :

**1-25 Crises généralisées ?**

- Oui
- Non

**1-26 Si crises généralisées, sont-elles ?**

- Atoniques
- Tonico-cloniques
- Myocloniques
- Toniques
- Cloniques

**1-27 Absences ?**

- Oui
- Non

**1-28 Crises avec début focale sans perte de connaissance ?**

- Oui
- Non

**1-29 Si crises avec début focale sans perte de connaissance, sont-elles ?**

- Motrices
- Sensorielles

**1-30 Crises avec début focal avec perte de connaissance ?**

- Oui
- Non

**1-31 Si crises avec début focal avec perte de connaissance, sont-elles ?**

- Motrices
- Sensorielles

**1-32 Crise avec début focal secondairement bilatéralement tonico-clonique ?**

- Oui
- Non

**1-33 Un agent de santé qualifié a-t-il confirmé au moins une fois un épisode de chutes/hochements de tête ou avez-vous un enregistrement vidéo d'une crise ?**

- Oui
- Non
- Inconnu

Développement psychomoteur de l'enquêté/comportement (comparativement aux enfants du même âge)

AVANT le début des chutes/hochements de la tête

**1-34 A quel âge le sujet a-t-il marché ?**
*Mettre l'âge en mois, 998 si jamais marché ou 999 si inconnu*

______________________

**1-35 A quel âge le sujet a commencé à parler avec des phrases ?**
*Mettre l'âge en mois, 998 si jamais parlé ou 999 si inconnu*

______________________

**1-36 Quel était son rendement scolaire ?**

- Bon
- Moyen
- Mauvais
- Médiocre
- Pas encore scolarisé
- Inconnu

**1-37 Quel était son comportement ?**

- [ ] Normal
- [ ] Agité
- [ ] Agressif
- [ ] Amorphe
- [ ] Mou (inconnu)

APRES le début des chutes/hochements de la tête

**1-38 Capacité de marcher ?**

- ○ Oui
- ○ Non
- ○ Inconnu

**1-39 Capacité de parler avec des phrases ?**

- ○ Oui
- ○ Non
- ○ Inconnu

**1-40 Quel est maintenant son rendement scolaire ?**

- ○ Toujours bon
- ○ Toujours moyen
- ○ En régression
- ○ Nul
- ○ Inconnu

**1-41 Quel est maintenant son comportement**

- [ ] Normal
- [ ] Agité
- [ ] Agressif
- [ ] Amorphe (mou)
- [ ] Errant (vagabond)
- [ ] Aliéné
- [ ] Inconnu

Paramètres anthropométriques

**1-42 Taille (cm)**
*Mettre 3 chiffres, si non prise mettre 999*

______________________

**1-43 Poids (kg)**
*Multiplier par 100 et mettre 3 chiffres, si non pris mettre 999*

______________________

**1-44 Périmètre brachial (cm)**
*Mettre 3 chiffres, si non pris mettre 999*

______________________

Examen neurologique

**1-45 Etat général**

- ◯ Bon
- ◯ Altere
- ◯ Grabataire

**1-46 Altération de la conscience ?**
*Des commentaires sur la conscience peuvent être faits à la fin du questionnaire si le neurologue le pense nécessaire*

- ◯ Normale
- ◯ Modérée
- ◯ Sévère

**1-47 Quelle est (était) la fréquence des chutes/hochements de tête ?**

- ◯ 1 à 5/mn
- ◯ 6 à 10/mn
- ◯ 11 à 15/mn
- ◯ > 15/mn
- ◯ Inconnu

**1-48 La fréquence a-t-elle augmentée avec l'évolution de la maladie ?**

- ◯ Oui
- ◯ Non
- ◯ Inconnu

**1-49 Quel (s) est (sont) le (s) facteur (s) déclenchant les chutes/hochements de tête ?**
*Plusieurs réponses possibles*

- ☐ Repas
- ☐ Froid
- ☐ Lumiere
- ☐ Bruit
- ☐ Au reveil
- ☐ Nuit
- ☐ Orages
- ☐ Autres
- ☐ Pas de facteur spécifique
- ☐ Inconnu

**1-50 Si autres, spécifier**

---

**1-51 Comment est la marche ?**

- ○ Normale
- ○ Base élargie
- ○ Spastique
- ○ Fauchante
- ○ Incapacité de marcher
- ○ Autre

**1-52 Si autre, préciser**

---

**1-53 Existe-t-il une faiblesse musculaire?**

- ○ Oui
- ○ Non

**1-54 Si oui, préciser**

- ○ Generalisee
- ○ Localisee

**1-55 Existe-t-il une paralysie?**

- ○ Oui
- ○ Non

**1-56 Si oui, préciser (localisation?)**

---

**1-57 Existe-t-il des contractures ?**

- Oui
- Non

**1-58 Existe-t-il un déclin cognitif ?**

- Oui
- Non

**1-59 Si oui, quelle fonction est touchée ?**

- Langage
- Mémoire
- Réalisation des gestes
- Reconnaissance des gens/objets
- Jugement
- Raisonnement

**1-60 Conclusion examen neurologique**

- Normal
- Anormal

**1-61 Si anormal, spécifier**

---

**1-62 Trouble ou retard mental ?**

- Oui
- Non

**1-63 Si oui, préciser l'importance :**

- Peu important (ne dérange pas les activités)
- Modérément important (dérange les activités mais encore actif)
- Important (empêche d'exercer les activités habituelles)

**1-64 Retard ou arrêt de croissance ?**

- Oui
- Non

**1-65 Retard sexuel (classification de Tanner)**

- ◯ Oui
- ◯ Non
- ◯ Enfance

**1-66 Si oui stade Tanner**

- ◯ Tanner 1
- ◯ Tanner 2
- ◯ Tanner 3
- ◯ Tanner 4
- ◯ Tanner 5
- ◯ Indeterminé

**1-67 Existe-t-il d'autres comorbidités ?**

- ◯ Oui
- ◯ Non

**1-68 Si oui, préciser :**

*Si certains examens antérieurs sont disponibles, on peut s'en servir*

- ☐ Anémie (clinique)
- ☐ Malnutrition sévère
- ☐ Convulsion à répétition rebelles au traitement
- ☐ Blessures multiples
- ☐ Plaies septiques
- ☐ Cicatrices chéloïdes
- ☐ Déformations articulaires
- ☐ Raideurs articulaires
- ☐ Autres

**1-69 Si autres, préciser**

---

**1-70 Déformations corporelles**

*Plusieurs réponses possibles*

- ☐ Thoracique
- ☐ Colonne vertébrale
- ☐ Membres
- ☐ Visage
- ☐ Autres
- ☐ Aucune

**1-71 Si autres, préciser**

______________________________________

**1-72 EEG fait ?**

- ◯ Oui
- ◯ Non

**1-73 Sinon pourquoi ?**

______________________________________

**1-74 Diagnostic**

- ◯ Syndrome du hochement de la tête
- ◯ Epilepsie sans hochement de tête
- ◯ Syndrome du hochement de la tête et autre épilepsie
- ◯ Non-épileptique
- ◯ Autre diagnostic

**1-75 Si Syndrome du hochement de la tête**

- ◯ Cas probable
- ◯ Cas confirmé
- ◯ Indéterminé

**1-76 Autre diagnostic à spécifier**

______________________________________

**1-77 Traitement fait ?**

- ◯ Oui
- ◯ Non

**1-78 Commentaires du Neurologue**

______________________________________
