# Supplementary material for: First description of Nodding Syndrome in the Central African Republic
Source: PLoS Negl Trop Dis. 2021 Jun 18;15(6):e0009430. doi: 10.1371/journal.pntd.0009430 (PMC8244846; doi:10.1371/journal.pntd.0009430)
Supplement: S4 File — (PDF) [file pntd.0009430.s004.pdf]

Nodding Syndrome en RCA 2019 (Histoire naturelle)

HISTOIRE NATURELLE DU NODDING SYNDROME

Nom de l'enquêteur

- ◯ Pascal MBELESSO
- ◯ Salvatore METANMO

Spécialité de l'enquêteur

- ◯ Neurologue
- ◯ Médecin généraliste

Date

yyyy-mm-dd
____

Identifiant

____

2-1 Situation matrimoniale :

- ◯ Marié (e)
- ◯ Union libre
- ◯ Célibataire
- ◯ Divorcé (e)/Séparé (e)
- ◯ Veuf (ve)

2-2 Avez-vous été à l'école?

- ◯ Oui
- ◯ Non
- ◯ Inconnu

2-3 Si Oui, dernière classe fréquentée

- CP1
- CP2
- CE1
- CE2
- CM1
- CM2
- 6ème
- 5ème
- 4ème
- 3ème
- 2nde
- 1ère
- Terminale
- Supérieur
- Inconnu

2-4 Quelle est votre occupation principale actuelle?

- [ ] Fonctionnaire ou Salarié (privé)
- [ ] Petit commerce
- [ ] Agriculture
- [ ] Pêche
- [ ] Chasse
- [ ] Elevage
- [ ] Artisanat
- [ ] Elève/Etudiant (e)
- [ ] Descolarisé (e)
- [ ] Inactif (ve)
- [ ] Enfant non encore scolarisé
- [ ] Autre

2-5 Si autre, préciser

____

2-6 Religion :

- ◯ Chrétienne
- ◯ Musulmane
- ◯ Autre

2-7 Si autre, préciser

2- 8 Ethnie :

Antécédents personnels

2-9 La mère est-elle là pour répondre aux questions concernant la grossesse/enfance?

- ◯ Oui
- ◯ Non

2-10 Lieu/structure de naissance de l'enquêté ?

- ◯ Domicile
- ◯ Centre de sante
- ◯ Hopital
- ◯ Autre
- ◯ Inconnu

2-11 Si autre, préciser

2-12 Accouchement

- ◯ Normal
- ◯ Accouchement difficile (ventouse/forceps)
- ◯ Césarienne
- ◯ Inconnu

2-13 Traumatisme à la naissance

- ◯ Oui
- ◯ Non
- ◯ Inconnu

2-14 Si oui, lequel ?

2-15 Le participant enquêté est-il né prématuré?

- ◯ Oui
- ◯ Non
- ◯ Inconnu

2-16 Si oui, terme de la grossesse (en semaines)

Mettre 99 si inconnu

____

2-17 Est-ce que l'enfant a crié aussitôt à la naissance ?

- ◯ Oui
- ◯ Non
- ◯ Inconnu

2-18 Avez-vous observé un des symptômes suivants avant le début des chutes de la tête ?

- ☐ Baisse de la compréhension
- ☐ Périodes de fixité ou de regard vide (évasif)
- ☐ Faiblesse corporelle générale
- ☐ Somnolence excessive
- ☐ Périodes d'innatention
- ☐ Léthargie (catalepsie, torpeur)
- ☐ Étourdissements (vertiges)

2-19 Si baisse de la compréhension, depuis quand ?

En mois, mettre 99 si inconnu

____

2-20 Si périodes de fixité ou de regard vide (évasif), depuis quand ?

En mois, mettre 99 si inconnu

____

2-21 Si faiblesse corporelle générale, depuis quand ?

En mois, mettre 99 si inconnu

____

2-22 Si somnolence excessive, depuis quand ?

En mois, mettre 99 si inconnu

____

2-23 Si périodes d'innatention, depuis quand ?

En mois, mettre 99 si inconnu

____

2-24 Si léthargie (catalepsie, torpeur), depuis quand ?

En mois, mettre 99 si inconnu

____

2-25 Si étourdissements (vertiges), depuis quand ?

En mois, mettre 99 si inconnu

____

2-26 Avez-vous observé un des symptômes suivants depuis le début des chutes de la tête ?

- [ ] Difficultés à parler (s'exprimer)
- [ ] Comportement agressif
- [ ] Épisodes répétés de regards vides
- [ ] Difficulté de concentration (Manque d'attention)
- [ ] Difficultés d'apprentissage
- [ ] Mémoire déficiente (troubles de la mémoire)
- [ ] Insomnies
- [ ] Difficulté d'endormissement
- [ ] Dort excessivement
- [ ] Hyperactif (agité)
- [ ] Amorphe (mou)
- [ ] Hallucinations visuelles
- [ ] Hallucinations auditives
- [ ] Déficience visuelle (baisse de la vision)
- [ ] Déficience auditive (baisse de l'audition)
- [ ] Errance (se perd et est recherché)
- [ ] Fugues (s'enfuit volontairement à plusieurs reprises)
- [ ] Périodes prolongées de mauvaise humeur ou de tristesse
- [ ] Mange mal (difficilement)
- [ ] Crises convulsives
- [ ] Déformations de la poitrine et du dos
- [ ] Déformations du visage
- [ ] Déformations des membres
- [ ] Invalidité grave
- [ ] Alité (grabataire)

2-27 Si difficultés à parler (s'exprimer), depuis quand ?
En mois, mettre 99 si inconnu

____

2-28 Si comportement agressif, depuis quand ?
En mois, mettre 99 si inconnu

____

2-29 Si épisodes répétés de regards vides, depuis quand ?
En mois, mettre 99 si inconnu

____

2-30 Si difficultés de concentration (manque d'attention), depuis quand ?
En mois, mettre 99 si inconnu

2-31 Si difficultés d'apprentissage, depuis quand ?
En mois, mettre 99 si inconnu

2-32 Si mémoire déficiente (troubles de la mémoire), depuis quand ?
En mois, mettre 99 si inconnu

2-33 Si insomnies, depuis quand ?
En mois, mettre 99 si inconnu

2-34 Si difficultés d'endormissement, depuis quand ?
En mois, mettre 99 si inconnu

2-35 Si dort excessivement, depuis quand ?
En mois, mettre 99 si inconnu

2-36 Si hyperactif (agité), depuis quand ?
En mois, mettre 99 si inconnu

2-37 Si amorphe (mou), depuis quand ?
En mois, mettre 99 si inconnu

2-38 Si hallucinations visuelles, depuis quand ?
En mois, mettre 99 si inconnu

2-39 Si hallucinations auditives, depuis quand ?
En mois, mettre 99 si inconnu

2-40 Si déficience visuelle (baisse de la vision), depuis quand ?
En mois, mettre 99 si inconnu

2-41 Si déficience auditive (baisse de l'audition), depuis quand ?
En mois, mettre 99 si inconnu

2-42 Si errance (se perd et est recherché), depuis quand ?
En mois, mettre 99 si inconnu

2-43 Si fugues (s'enfuit à plusieurs reprises), depuis quand ?
En mois, mettre 99 si inconnu

2-44 Si périodes prolongées de mauvaise humeur ou de tristesse, depuis quand ?
En mois, mettre 99 si inconnu

2-45 Si mange mal (difficilement), depuis quand ?
En mois, mettre 99 si inconnu

2-46 Si crises convulsives, depuis quand ?
En mois, mettre 99 si inconnu

2-47 Si déformations de la poitrine et du dos, depuis quand ?
En mois, mettre 99 si inconnu

2-48 Si déformations du visage, depuis quand ?
En mois, mettre 99 si inconnu

2-49 Si déformations des membres, depuis quand ?
En mois, mettre 99 si inconnu

2-50 Si invalidité grave, depuis quand ?
En mois, mettre 99 si inconnu

____

2-51 Si alité (grabataire), depuis quand ?
En mois, mettre 99 si inconnu

____

2-52 L'enquêté prend t-il ou a-t-il pris des médicaments depuis le début des crises (HT) ?

- ◯ Oui
- ◯ Non
- ◯ Inconnu

2-53 Si oui, à quelle fréquence ?

- ◯ De façon régulière (périodicité)
- ◯ Apres chaque crise
- ◯ Continuellement
- ◯ Inconnu

2-54 Quel(s) médicament(s)

- ☐ Anti-convulsivant
- ☐ Anti-épileptique
- ☐ Médicament traditionnel
- ☐ Autre

2-55 Si autre, préciser

____

2-56 L'enquêté a-t-il pris de l'ivermectine ou Mectizan (par voie orale) ?

- ◯ Oui
- ◯ Non
- ◯ Inconnu

2-57 Si oui avant et/ou après le début des crises ?

- ◯ Avant
- ◯ Après
- ◯ Avant et Après
- ◯ Inconnu

2-58 L'enquêté prend t-il ou a-t-il pris d'autres médicaments sur une période supérieure à 3 mois ?

- ○ Oui
- ○ Non
- ○ Inconnu

2-59 Si oui, quel médicament ?

Habitudes alimentaires

2-60 L'enquêté consomme-t-il régulièrement de la viande ?

- ○ Oui
- ○ Non
- ○ Inconnu

2-61 Si oui, quelle viande consomme t'il fréquemment ?
Remarque : On peut citer plusieurs viandes, mettre "Inconnu" si ne sait pas

---

2-62 L'enquêté consomme t'il régulièrement le poisson ?

- ○ Oui
- ○ Non
- ○ Inconnu

2-63 Si oui, quelle poisson consomme t'il fréquemment ?
Remarque : On peut citer plusieurs poissons, mettre "Inconnu" si ne sait pas

---

2-64 Quels autres aliments consomme-t-il régulièrement ?

- ☐ Légumes
- ☐ Fruits
- ☐ Produits laitiers
- ☐ Tubercules
- ☐ Fruits de mer
- ☐ Oeufs
- ☐ Autres

2-65 Si autres, préciser

2-66 Quelle eau consomme-t-il régulièrement ?

- ◯ Réseau publique (eau courante)
- ◯ Forage
- ◯ Minérale
- ◯ Rivière
- ◯ Puits
- ◯ Autre

2-67 Si autre, préciser

Antécédents familiaux

2-68 L'enquêté a combien de frères et sœurs ?
Mettre 99 si inconnu

____

2-69 Quel est sa position dans la fratrie ?
Mettre 99 si inconnu

____

2-70 L'enquêté a-t-il un jumeau ou une jumelle ?

- ◯ Oui
- ◯ Non
- ◯ Inconnu

2-71 Si oui, a-t-il (elle) aussi des chutes de la tête ?

- ◯ Oui
- ◯ Non
- ◯ Inconnu

2-72 Est-ce qu'il y a d'autres personnes avec chute de la tête dans la famille ?
En dehors du jumeau le cas échéant

- ◯ Oui
- ◯ Non
- ◯ Inconnu

2-73 Si oui, est-elle décédée ou vivante ?

- ○ Décédée
- ○ Vivante
- ○ Inconnu

2-74 Y a-t-il des cas d'épilepsie dans la famille ?

- ○ Oui
- ○ Non
- ○ Inconnue

2-75 Y a-t-il eu des pénuries alimentaires dans la famille ?

- ○ Oui
- ○ Non
- ○ Inconnu

Histoire de la migration

2-76 Depuis quand le participant vit-il dans le village?

- ○ < 1 an
- ○ > 1 an
- ○ Inconnu

2-77 Le participant a-t-il vécu dans un autre village ?

- ○ Oui
- ○ Non
- ○ Inconnu

2-78 Si oui, lequel?

2-79 En quelle année s'est-il déplacé ?
Mettre 999 si inconnu

2-80 Pour quelle raison s'est-elle déplacée?

- ○ Guerre
- ○ Raison professionnelle (même d'un autre membre de la famille)
- ○ Autre
- ○ Inconnu

2-81 Si autre, préciser

2-82 Avez-vous vécu dans des camps de réfugiés (avec la famille) ?

- Oui
- Non
- Inconnu

2-83 Avez vous connu et vécu la guerre ?

- Oui
- Non
- Inconnu

2-84 L'enquêté a-t-il subit des traumatismes de guerre avec séquelles psychologiques ?

- Oui
- Non
- Inconnu

2-85 Acceptez vous qu'une photo soit prise ?

Click here to upload file. (< 5MB)

2-86 Acceptez vous qu'une 2ème photo soit prise ?

Click here to upload file. (< 5MB)

2-87 Commentaires libres de l'enquêteur
