# Supplementary material for: First description of Nodding Syndrome in the Central African Republic
Source: PLoS Negl Trop Dis. 2021 Jun 18;15(6):e0009430. doi: 10.1371/journal.pntd.0009430 (PMC8244846; doi:10.1371/journal.pntd.0009430)
Supplement: S5 File — (PDF) [file pntd.0009430.s005.pdf]

Clinical description of possible cases of Nodding Syndrome excluded from paper

Case N° 6: Male, aged 9 years, unschooled, with a history of tuberculous meningitis with temporary tonic-clonic seizures at 4 years of age. Unprovoked convulsive seizures started at 6, and nodding seizures at 7. Nodding seizures were triggered by noise and heat, and occurred at a frequency of 4 HN per minute. Several other symptoms developed thereafter: aggressive behavior, repetitive starring episodes, difficulties concentrating, wandering, and auditory and visual hallucinations especially while sleeping.

The neurological examination revealed a psychomotor agitation, deafness and muteness, mild mental impairment, bulimia, cognitive decline associated with language deficits, impaired judgment and reasoning.

Case N° 7: Female, aged 9 years, unschooled, with nodding seizures since the age of 6. She had reportedly been subject to severe acute malnutrition (SAM) as early as when she was 6 months old, leading to a delayed psychomotor development with severe hypotrophies which were still observable at the time of the study. During this period of SAM (66 months prior to nodding seizures onset), several symptoms had developed and persisted to date, including decreased learning and comprehension capacities, attention deficits, blank starring episodes, difficulties concentrating, and auditory deficits.

This child was emaciated, severely underfed, with an evasive look and clinical malnutrition (brittle brownish hair). She had never developed spoken language, had cognitive decline affecting her memory, language and executive functions. We equally noted moderate mental retardation, an ataxic gait, and spinal deformity. Nodding seizures in this patient were triggered by fasting, and often followed by generalized tonic-clonic seizures.
