# Supplementary material for: First description of Nodding Syndrome in the Central African Republic
Source: PLoS Negl Trop Dis. 2021 Jun 18;15(6):e0009430. doi: 10.1371/journal.pntd.0009430 (PMC8244846; doi:10.1371/journal.pntd.0009430)
Supplement: Attachment — Submitted filename: Response to reviewers.docx [file pntd.0009430.s006.docx]

**Reviewers' comments**

**Methods**

**Reviewer #1**

**Comment:**

The region studied was a convenience sample in the sense of accessibility, but within the population studied this was a household survey and well described. Screening was done using a series of screening questionnaires and finally a review by a neurologist to decide the definitive diagnosis. Copies of the questionnaires are in the supplementary material. For what is effectively a preliminary study, this was adequate. To draw definitive conclusions it would be best to expand the surveyed area but as a first study this is adequate. Ethical requirements were met.

**Response:**

We thank the referee for this comment. We plan to carry out a larger scale study in order to draw the appropriate conclusions.

**Reviewer #2**

**Comment:**

The manuscript fulfills all of the requirements for Methods as listed above.

**Response:**

We thank the referee for this comment.

**Reviewer #3**

**Comment 1:**

The paper by Metanmo et al. presents the investigation of epileptics in the Central African Republic to describe cases of Nodding Syndrome. Their paper reports an extremely low prevalence of NS, but still with 5 cases described. Probably because of the very low number of cases, the authors then presented the paper in a more clinical manner, describing the history of the disease and the clinical characteristics of the patients.

**Response 1:**

We thank the referee for this comment and he/she is right: we presented the paper in a clinical manner because the number of reported NS cases was low.

**Comment 2:**

The authors conducted a cross-sectional community-based study in one village (Landja) and applied a door-to-door approach. The authors should clarify if they surveyed systematically all the households in the selected villages. If this was the case, the study will provide an exhaustive census of the epileptic subjects and NS cases. The authors just say “each household” (line 134), but to be precise, the authors should add in the Methods the exact number of households targeted (from a census, for example) with the number of inhabitants targeted.

The strength of the paper is that the study has been able to carry out all stages of examinations, including examinations by neurologists and the performance of EEGs. Moreover, the authors have presented well the limitations of the study, especially regarding the problems of classifying the disease.

**Response 2:**

We thank the referee for this comment. All the households of the village (Landja) in CAR were investigated. We changed "each household" to "all the households" in the manuscript. No census data were available before the investigation in the village because administrative and demographic data are missing in this war-affected country.

**Comment 3:**

The clinical descriptions are complete, well presented and interesting.

**Response 3:**

We thank the referee for this comment.

**Comment 4:**

Please, the authors should add information regarding the “active onchocerciasis transmission” status in the study setting (line 116), and add a specific point in the Discussion on the fact that they did not find more NS cases in this seemingly active onchocerciasis transmission setting. Information on nodule prevalence would be useful. In other words (lines 133-134): how do the authors explain that there are no data on *Onchocerca volvulus* endemicity when they state that this is?

**Response 4:**

No data on *Onchocerca volvulus* endemicity were available in this area at the time of the study (December 2019). In January 2020, we conducted an Ov16 antibody prevalence study of onchocerciasis in the same area among children, aged 6–9 years. The overall seroprevalence was 8.9%, suggesting that there is an ongoing *Onchocerca volvulus* transmission in the Landja area, but the endemicity of onchocerciasis in the area is low (de Smet et al, 2019).

This point was clarified in the manuscript and we added a specific point in the Discussion section on the fact that we found only 5 NS cases.

**Comment 5:**

Lines 105-106: Please, add a reference to substantiate this sentence, because it is important to highlight previous reports on possible NS cases in CAR.

**Response 5:**

This information has not been the subject of any scientific publication. In the First International Workshop on Epilepsy Associated with Onchocerciasis held in Antwerp (Belgium) from 12 to 14 October 2017 (Colebunders et al, 2018), we were told informally by colleagues from CAR that they suspected NS cases near Bangui. We added a reference to the workshop in the manuscript.

This point was clarified in the manuscript in the introduction section.

**Comment 6:**

Mental retardation could be more explained.

Please see below: ‘Mental retardation’ has largely been supplanted with a less stigmatizing term, such as ‘developmental and cognitive regression’. Please use the latter term instead of Mental retardation.

**Response 6:**

We thank the referee for this suggestion. After discussing this issue with the neurologists involved in this study, we suggest the following consensual sentence: “developmental delay and cognitive decline”

We have replaced the term "mental retardation" with “developmental delay and cognitive decline” throughout the text and in the tables.

**Results**

**Reviewer #1**

**Comment:**

Yes. Results are well presented and the tables complete and informative.

**Response:**

We thank the referee for this comment.

**Reviewer #2**

**Comment:**

The Results section analysis plan and the analysis presentation match. The tables and figures are adequate.

**Response:**

We thank the referee for this comment.

**Reviewer #3**

**Comment 1:**

The results are well presented and correspond to the proposed Methods. They are, however, essentially clinical descriptions, not based on statistical evaluations.

**Response 1:**

We thank the referee for this comment. Due to the small sample size, no statistical evaluations were performed.

**Comment 2:**

However, the authors should present the percentage of the population evaluated in relation to the target population (see problems in the Methods highlighted above).

**Response 2:**

We thank the referee for this comment. As mentioned above, we have no data on the actual size of the population other than what we collected ourselves (6,175). This is therefore the denominator in the calculation of our prevalence (0.8 per 1,000).

**Comment 3:**

Line 186: 5 NS cases among 55 PWE: 9.1% and not 8.3%? Please clarify this percentage, or correct it if there is a mistake. If this is a mistake, please, check all your values throughout the manuscript.

**Response 3:**

The denominator for this calculation is 60, which represents the number of PWE, i.e. 55 cases of non-NS epilepsy and 5 cases of NS.

**Comment 4:**

Although the authors focused on NS, results on retarded growth and mental retardation may be included for all this population.

**Response 4:**

We thank the referee for this suggestion. As emphasized by this reviewer, we chose to focus on NS cases in this article.

**Comment 5:**

Please see below: ‘Mental retardation’ has largely been supplanted with a less stigmatizing term, such as ‘developmental and cognitive regression’. Please use the latter term instead of Mental retardation.

**Response 5:**

We thank the referee for this comment. As mentioned above, we used the less stigmatizing term “developmental delay and cognitive decline” throughout the revised manuscript.

**Conclusion**

**Reviewer #1**

**Comment:**

Because only 5 probable cases were found, numbers are not high but considering this is an area that has not been described before, the conclusions are valid within the limits of the survey. This is of considerable public health importance although the pathogenesis of nodding syndrome is still poorly understood. The conclusion that the area needs more study is certainly valid and from the public health point of view, the area needs an epidemiological evaluation of onchocerciasis urgently which could have been mentioned in the text.

**Response:**

As mentioned above, no data on *Onchocerca volvulus* endemicity were available in this area at the time of the study (December 2019). In January 2020, we conducted an Ov16 antibody prevalence study of onchocerciasis in the same area among children, aged 6–9 years. The overall seroprevalence was 8.9%, suggesting that there is an ongoing *Onchocerca volvulus* transmission in the Landja area, but the endemicity of onchocerciasis in the area is low (de Smet et al, 2019).

This point was clarified in the manuscript and we added a specific point in the Discussion section on the fact that we found only 5 NS cases.

**Reviewer #2**

**Comment:**

The conclusions are supported by the data. The limitations, while clearly described, are insufficient and require some additional work. There is no comment on public health relevance or an extrapolation on how the data can be useful in our overall understanding of the topic.

**Response:**

We thank the referee for this comment. We have added a sentence on public health relevance in the conclusion:

“NS is a public health problem in affected areas, but the disease is not yet known in CAR and therefore not managed efficiently. It is therefore important to sound the alarm at the level of the local health authorities in order to implement adequate care for these children.”

We added an additional paragraph about the limitations of the study which is related to the questionnaires. The new paragraph reads as follows:

“To our knowledge, validated questionnaires for screening and confirmation of NS cases are not available. We therefore used non-validated questionnaires that were inspired by all the studies carried out by experts in the field. There was a variability between interviewers during the screening phase because the investigators were organized in three teams but all interviewers were trained before the study, had the same degree of education, and asked only multiple choice questions. The two confirmation questionnaires and the EEG questionnaire were administered by the same team, thus eliminating inter-interviewer variability.”

**Editorial and Data Presentation Modifications?**

**Reviewer #1**

**General Comments to the authors:**

Although NS has not been described before in CAR, epilepsy has been. It would be good to include a reference to this in the background information.

**Response:**

We thank the referee for this suggestion and have added it in the introduction section.

Limited data are available on epilepsy in CAR, particularly in the study area. The most recent data showed a prevalence of epilepsy of 2.8‰ in Bangui (Mbelesso et al, 2009). This reference was added in the manuscript in the introduction section.

**Specific Comments to authors:**

**Comment:**

Line 45: We identified 5 "probable" cases of which one was certified NS according to the WHO definitions. This is what the survey showed but it is a bit confusing as written in the Abstract.

**Response 1:** We thank the referee for this remark. We clarified this point in the abstract.

Line 63: I would add "since its first "described" appearance 60 years ago

**Response 2:** Done

Line 65: "and" psychiatric

**Response 3:** Done

Line 165: How do the CDC figures relate to the CAR population?

**Response 4:** We used these CDC figures because they were already used in Cameroun (which is a neighboring country of CAR) for investigating NS (Siewe et al 2019). The CDC figures were also used in another recent study in Uganda in 2018 (Kaiser et al 2018).

Line 181: The seven cases identified were based on what level of survey. Two were excluded later. Who made these decisions based on what?

**Response 5:** Several neurologists are part of the team (PM, ASW, PMP). Not all of them were in the field, but after discussions between them, they all agreed that it would be better to remove these two cases mainly because of their history (see details in supplementary file 5). They could be “NS mimic” such as partial or generalized complex atonic seizures. As the definition of NS is not clear, all agreed that these two cases could be considered as NS cases in the future, according to the evolution of knowledge in the field.

Line 221: started "in" 2019

**Response 6:** Done

Line 315: Physical deformities of the limbs needs more description. My impression from the text is that these were functional deformities of the lower limbs, not physical ones.

**Response 7:** These are physical deformities leading to functional disorders such as severe steppage. This deformity was best described in case 1 (line 199 - 200) and the new sentence reads as follows:

“Left inwardly turned foot, tendon retractions of the two lower limbs more severe on the left, and stumbling gait”

Line 330: Is this just Central Africa or the Central African Republic? Needs correction.

**Response 8:** This is Central Africa (Cameroon and CAR). The repetition has been removed and we clarified this point in the revised manuscript.

Line 354: Does the 80% refer to all patients with epilepsy or just NS? 80% of a sample of 5 is stretching percentages a little!

**Response 9:** This is the sample of the 5 NS cases. The term 80% has been removed and replaced by "four".

Line 372: Could be an overestimation as well as an underestimation.

**Response 10:** We thank the referee for this remark and we agree. We deleted “and probably represent an underestimation” in the revised manuscript.

Line 382: first description of "probable" NS in CAR

**Response 11:** Done

**Reviewer #3**

**Comments:**

- Check for missing spaces (as in line 341 with reference 2)

**Response 1:** Done

- Clarify affiliation 3: which unit of the IRD?

**Response 2:** U1094 (https://lab.ird.fr/partenaire/3108/show)

- Harmonise references: e.g. ref 4 (the month is not indicated), and ref 5 (the month of publication is indicated). Please use PLoS NTDs guidelines for preparation of References.

**Response 3:** Done

**Summary and General Comments**

**Reviewer #1**

**Comment:**

This was an important survey and establishes the probability of NS in CAR as well as epilepsy. It definitely needs to be extended to the larger area. I would like to have seen some information on onchocerciasis prevalence in the paper. With the REMO mapping this was not considered an area of importance, perhaps because at that time it was not populated. Were these old well established communities or were they new communities?

**Response:**

As mentioned above, no data on *Onchocerca volvulus* endemicity were available in this area at the time of the study (December 2019). In January 2020, we conducted an Ov16 antibody prevalence study of onchocerciasis in the same area among children, aged 6–9 years. The overall seroprevalence was 8.9%, suggesting that there is an ongoing *Onchocerca volvulus* transmission in the Landja area, but the endemicity of onchocerciasis in the area is low (de Smet et al, 2019).

This point was clarified in the manuscript and we added a specific point in the Discussion section on the fact that we found only 5 NS cases.

For the largest part of the population, this was a well-established community.

**Reviewer #2**

**Comment:**

PNTD-D-20-02016

First Description of Nodding Syndrome in the Central African Republic

**Response:** Done.

**Comments to Authors:**

In this manuscript, the authors report an observational cross-sectional descriptive study of a clinical syndrome consistent with Nodding Syndrome (NS) in the Central African Republic (CAR). They describe the results of a community-based survey, during which they identify a number of persons with epilepsy, and identify a small subset of these persons whom, they state, suffer signs and symptoms consistent with NS. This would represent the first description of NS in CAR, and one of the only descriptions of the syndrome outside of East Africa.

Given that the substantive evidence that what the authors are observing / describing is in fact NS, the authors are wise to list Pros and Cons arguing for and against NS in this manuscript. Only one of the 5 described cases was ‘confirmed’ per WHO case classification criteria, while the other 4 described were considered ‘probable’. Although this does speak of the difficulty in documenting head nodding by witness in these cases, and the challenges of ‘confirming’ an event that is intermittent and paroxysmal, nonetheless it would be more reassuring and convincing if there were more observations of the head nodding behavior, to ensure that the behavior noted was in fact NS, and not a NS ‘mimic’ such as partial complex or generalized atonic seizures.

**Response:** We thank the referee for this comment. We clarified the state of the art of NS in Africa in the background section.

**Specific Comments and Questions:**

1. Lines 103-105 – ‘…the only significant association was found in certain areas with onchocerciasis or river blindness…’. This statement is not entirely true. Investigations have found a small, but significant association with being seropositive for *Onchocerca* infection. And, only in limited areas (e.g., there is plenty of onchocerciasis in areas in which NS, or for that matter higher incidence of epilepsy, is not found). The focus on onchocerciasis, in the absence of any data in the manuscript about the situation of onchocerciasis in this area of CAR (e.g., whether it is endemic / hyperendemic; the proportion of persons infected with *Onchocerca volvulus*) is another attempt by some of the authors on the manuscript to push forth their hypothesis on the relationship between *O. volvulus* and epilepsy in general, and NS specifically.

**Response 1:** We thank the referee for this comment and edited the sentence accordingly ("Even though the cause of this syndrome remains unclear, an association was found in several areas with onchocerciasis or river blindness due to the parasite *Onchocerca volvulus* (Line 103-105) "). Furthermore, additional information on the status of onchocerciasis in the study area has been included in the discussion section (“*Onchocerciasis endemicity*”).

2. Lines 137-138 – the authors state that the initial screening questionnaire was piloted in healthy volunteers…but without performance validation’. This is disturbing. The authors screened their population in order to get to the lowest denominator using an instrument that was not assessed for its sensitivity and specificity to identify epilepsy / seizures. This deserves a comment.

**Response 2:** We thank the reviewer for this remark. To our knowledge, there is no validated questionnaire for screening NS cases. We have used the work already done in the literature and by experts in the field to draw up a non-validated questionnaire that can be used in a survey on NS cases. We have revised this point in the "limitations" section, as follows:

“To our knowledge, validated questionnaires for screening and confirmation of NS cases are not available. We therefore used non-validated questionnaires that were inspired by all the studies carried out by experts in the field”.

3. The use of no fewer than 4 individual questionnaires is worth comment as well. How did the investigators ensure minimum inter-observer /inter-evaluator variability? How were all these forms piloted?

**Response 3:** We thank the reviewer for this remark and also added this element in the "limitations" section as follows:

“There was a variability between interviewers during the screening phase because the investigators were organized in three teams but all interviewers were trained before the study, had the same degree of education, and asked only multiple choice questions. The two confirmation questionnaires and the EEG questionnaire were administered by the same team, thus eliminating inter-interviewer variability.”

4. Line 166 and throughout – ‘Mental retardation’ has largely been supplanted with a less stigmatizing term, such as ‘developmental and cognitive regression’. Please use the latter term instead of Mental retardation.

**Response 4:** After discussing this issue with the neurologists involved in this study, we suggest the following consensual sentence: “developmental delay and cognitive decline”

5. Results, Line 179 – I’m a bit confused about the population being assessed; the total interviewed was 6,175 out of a total population of over 35,000? More detail needs to be provided as to how households were chosen / persons enrolled.

**Response 5:** The village (Landja-mboko) is divided into 6 districts. As mentioned above, no census data were available before the investigation in the village because administrative and demographic data are missing in this war-affected country. We investigated the first district (Landja) because it was the closest to Bangui and the easiest to access. We have no data on the actual size of the population in the first district other than what was collected ourselves (6,175).

6. I’m unclear why the child with severe acute malnutrition and HN was excluded from assessment. Is it not likely that many of these children, perhaps also those demonstrating HN, also suffered from severe malnutrition?

**Response 6:** Indeed, malnutrition was described in some children in East Africa as a risk factor of NS. But it is unclear whether malnutrition precedes the onset of NS or is a consequence of the disease, particularly in our study. The neurologists involved in this study preferred to remain cautious about this case and decided not to consider it as a probable case of NS.

7. Case 1 – if the child was witnessed to have HN only for 30 seconds, I don’t know how reliably this finding could be extrapolated out to 1 full minute…

**Response 7:** We witnessed the HN during 30 seconds. But similarly with the other affected children, these crises were also described by the family as being longer on many occasions.

8. It’s a bit concerning that the one EEG that was conducted during an actual nodding episode was without electrographic correlate…

**Response 8:** We agree with the concern of the reviewer. There is a possibility that the head nodding was not a symptom of NS or was indeed a symptom of the disease but the EEG was not able to pick it up because of the shortness of the crisis. However, this fact was noted in the arguments against NS.

9. While the WHO criteria are challenging to meet for confirmed cases, this is what was intended when the criteria were developed. Particularly in an area in which an attempt is being made to substantiate whether NS occurs / exists in the area, it is important to directly observe the head nodding episodes, and ideally in more than one subject. Once head nodding is substantiated to be occurring, then the criteria can be loosened a bit.

**Response 9:** We thank the referee for this suggestion and we agree. We will take this comment into account when we carry out a larger-scale study in CAR.

10. The fact that nearly 40% of the cases identified in the screening process were not evaluated by a neurologist is also concerning, and suggests that there might have been an insufficient number of patients with head nodding to differentiate other forms of seizures from ‘head nodding’.

**Response 10:** We thank the referee for this comment. This point was stressed in the section pointing out the limitations of the study.

**Reviewer #3**

**Comment:** In conclusion, this MS could form the basis for a possible publication. But is this really the first time that cases of NS are reported in CAR? Published perhaps, but reported? and the paper requires further discussion on the very low number of NS while the authors indicate that it is an active transmission area for onchocerciasis.

**Response:** As mentioned above, we modified the discussion accordingly.
